# Wessex Branch of the Association of Clinical Pathologists, Meeting at Bristol, December 1988

**Published:** 1990-03

**Authors:** 


### Wessex Branch of the Association of Clinical Pathologists

### Meeting held at Southmead Hospital, Saturday 10 December 1988

**The 1988 annual Winter Meeting of the Wessex branch of the association of Clinical Pathologists was held at the Postgraduate Medical Centre of Southmead Hospital on Saturday 10 December 1988 under the Presidency of Professor P P Anthony (Exeter). The organisation for this day meeting had been most effectively carried out by Dr Elizabeth Mackenzie (Southmead), the new Wessex branch secretary. The meeting was also attended by the national Chairman of Council, Dr B Murphy (Torbay), and the new President of the Association of Clinical Pathologists, Dr J Burston (formerly of Portsmouth) who is now resident in Cornwall. Drs G Russell (Bristol Children's Hospital) and A P MacGowan (Southmead) were elected as Junior Representatives to the Association. During the Scientific Session the papers listed below in abstract form were amongst those presented. About 50 members and guests participated.**

**In more stable times, the deliberations of wider political nature on the part of the Association are not usually reported in these columns. However, national politics are now clearly intruding on hospital laboratory medicine, and it should be said that matters of immediate and future concern to pathologists in all disciplines were discussed at the formal meetings of the ACP Branch and of the regional members of the Royal College of Pathologists. The potentially serious problems of laboratory management, the effects of the changes anticipated in 1992 and possible bids for privatisation of certain pathological services were given a full airing. The present and future disastrous effects of budgetary constraint, salary levels and career structure on our Medical Laboratory Scientific Officers were also given very detailed consideration. The issues outlined in this paragraph are of great concern to all who are attempting to provide pathology services. It was agreed that there are many issues which require solutions that are not properly addressed at the moment.**

**The next meeting in Spring 1989 of the Wessex branch of the Association of Clinical Pathologists will take place in Bath in conjunction with the Mid Southern branch.**

### A STUDY OF THYROID FUNCTION IN PREMATURE NEONATES

**A McConnell**
Southmead Hospital, Bristol

A longitudinal pilot study of thyroid function in 35 premature (gestation earlier than 32 weeks) neonates was carried out in order to investigate the correlation between respiratory outcome, especially bronchopulmonary dysplasia, and thyroid status. Commercial kits were used to measure plasma concentrations of free $T_3$, free $T_4$, total $T_4$ and TSH, while an in-house method was employed for measurement of thyroid-hormone-binding-globulin concentration. Samples were obtained from cord blood and from venous blood taken at day 1, 3, 5, 7, 14 and 28 of life. Since all sample volumes were small, the commercial kits were used at half-volumes and analyses performed in singlicate.

On the basis of the entire clinical history at discharge, the infants were classified as belonging to one of the following groups: (1) those who had minimal respiratory problems—the MRP group, numbering 6 infants; (2) those who developed the respiratory distress syndrome—the RDS group, numbering 16 infants; and (3) those who developed bronchopulmonary dysplasia—the BPD group, numbering 13 infants.

One of the most striking features of this study is the low level of free $T_3$ concentrations in all three groups; even at day 28 of life the mean $T_3$ concentrations were well below the adult reference range. The mean values of the concentrations of free $T_4$, total $T_4$, TSH and thyroid-hormone-binding-globulin for each of the three groups were normal. As a means of predicting which infants were most at risk of developing bronchopulmonary dysplasia, measurement of thyroid hormones adds little; combining free $T_4$ concentration at day 1 of life, the most discriminating of the thyroid parameters in this study, with birth weight alone as a predictor of the probability of developing bronchopulmonary dysplasia.

### NEEDLESTICKS–LIFE AT THE SHARP END

**M J Wilson**
Southmead Hospital, Bristol

There are many reports of needlesticks in the literature. Infections have been transmitted in volumes of blood as small as one microlitre, both by direct patient contact and indirect contact in the laboratory.

The risk of HIV infection following a needlestick involving an HIV positive patient is relatively small (estimated at $0.1-1\%$). However, there is no satisfactory treatment and the prevalence of HIV is increasing. The risk of hepatitis B after a positive needlestick is as much as 26% for hep B e Ag positive patients (less than 10% if e Ag negative).

It is difficult to assess the incidence of needlesticks in doctors due to under-reporting. Under-reporting is due to the apparent triviality of a needlestick and the nuisance of follow-up.

Needlesticks are particularly common in the inexperienced. Training should primarily be aimed at students beginning their clinical course. Education should be clear and concise, it should emphasize the importance of safe venepuncture and needle disposal.

Unsafe resheathing accounts for the majority of needlesticks among doctors. Replacing a needle into a hand held sheath accounts for injuries to the finger tips. These injuries may be avoided by replacing a needle into a sheath on the edge of a flat surface. Needleguards such as "The vacutainer mushroom" are safe but many junior doctors consider them clumsy. Alternatively, the needle and syringe can be placed directly into a sharps bin without resheathing.

Sharps bins should be sturdy and strategically placed. They should not be over filled and they should be carefully sealed.

I have interviewed 53 junior doctors at Southmead Hospital. The discussions were in confidence and also related to other aspects of phlebotomy. Forty-two doctors resheath unsafely. Thirty-six doctors have had needlestick (twelve have had multiple needlesticks), only six doctors have had needlesticks followed up.

These figures are unacceptable. It is hoped that thorough training in safe venepuncture and disposal will reduce the incidence of needlesticks in the future.

### GENETIC ANALYSIS OF DIFFERENT KINDS OF PHENYLKETONURIA IN SEVERAL AFFECTED MEMBERS OF ONE FAMILY

**Linda Tyfield**
Southmead Hospital, Bristol

It has been known for some time now that there are several different kinds of phenylketonuria (PKU) but it has only been recently, with the availability of a specific gene probe, that it has become possible to correlate these differences with specific mutations at the level of the gene.

We have investigated a family in which there are two affected children who are first cousins who had different biochemical presentations of PKU in the neonatal period. Using a full length cDNA probe we have defined the haplotype patterns of the mutant PKU genes in these children. Through gene tracking we have shown that they both inherited the same mutant gene from their grandfather who also has PKU which was previously undiagnosed. He is of normal intelligence but he has never had dietary phenylalanine restriction. All affected members have a different haplotype pattern on their second mutant allele. We are attempting to correlate these genetic differences with biochemical and clinical status.

### DIAMONDS AND ELASTIN—DIAGNOSTIC AIDS IN SOLITARY RECTAL ULCER SYNDROME

**B F Warren, E K Dankwa, J D Davies**
Bristol Royal Infirmary

The diagnosis of prolapsing rectal mucosa syndrome and solitary rectal ulcer syndrome in an endoscopic biopsy can present difficulty. We report previously unrecognised features of these conditions which we find helpful.

Thirty-two rectal biopsies with a histological diagnosis of either of the above conditions were reviewed to look at the frequency of occurrence of diamond-shaped crypts. The associated thickening, separation and disruption of the muscularis mucosae was also noted. Each biopsy was also stained with elastic van Gieson.

Thirty biopsies included frequent diamond-shaped crypts. The muscularis mucosae was thickened and disrupted, and vertical muscle fibres were seen in the lamina propria in all biopsies. These features did not differ between the two diagnoses. Ulceration was seen in two solitary rectal ulcer syndrome cases. Inflammation varied widely in amount. The amount of elastin was increased and elastin fibres splayed out from the surface of the muscularis mucosae to ensheath diamond-shaped crypts in thirty cases.

All cases had either diamond-shaped crypts or pericrypt elastin; both features were seen in 28 cases.

We conclude that crypts with pointed, diamond-shaped bases and an increased ensheathing elastin pattern are constant and helpful diagnostic features of these syndromes.

### THE COLONOSCOPIC ASSESSMENT OF CHRONIC ULCERATIVE COLITIS—HOW MANY BIOPSIES ARE NECESSARY?

**H S Rigby, B F Warren, C Neumann, R A Mountford, J W B Bradfield**
Bristol Royal Infirmary

The technique of multiple colonoscopic biopsies is used in chronic ulcerative colitis to confirm extent and severity of disease and to assess dysplasia. Flexure biopsies were introduced in the 1970s but their contribution has never been properly audited. This study, therefore, audits the contribution of flexural biopsies in the assessment of longstanding ulcerative colitis in 72 biopsies. A histological disease activity index (HDAI) was devised and the inflammation in each biopsy scored on a 0–6 scale. Sixty-one of the reports included biopsies of the splenic flexure and both adjacent sites. In 56 of these biopsies the assessment made did not differ from that from the adjacent sites. Five of the biopsies differed from both adjacent sites by a score of only one. Three of the splenic flexure biopsies showed dysplasia, but this was never seen at this site alone. Out of 51 hepatic flexure biopsies with adjacent sites 49 contributed nothing extra. Two differed from both adjacent sites, one by a score of one and the other by a score of two. Dysplasia was never seen at the hepatic flexure. We conclude that the addition of flexural biopsy contributes nothing to the management of longstanding total ulcerative colitis and their continued use is questioned.

### LISTERIOSIS IN BRISTOL

**A P MacGowan**
Southmead Hospital, Bristol

Twenty-two cases of Listeriosis occurred in Bristol between January 1983 and November 1988. The incidence of infection was 0.44 cases/year/$10^5$ population. Fifty per cent (11 cases) were pregnancy associated or in newborns giving an incidence of one case per 4,955 live births. On average there were 3.2 cases/year between 1983 and 1987, but 6 cases occurred in 1988. Seven (32%) cases occurred between January and June, but the majority (68%) were in the months between July and November. Of the non-pregnancy and newborn cases the mean age was 61 years (range 6–81) and 82% (9 cases) were immunosuppressed: 4 patients had haematological malignancies; 4 solid tumours; 2 renal transplants; 9 were being treated with steroids and 5 cytotoxic drugs. Four of the adult cases presented with meningitis and seven bacteraemia. The mortality was 36% amongst the adults and children. Antibiotic therapy was variable but five patients received an aminoglycoside, six ampicillin or penicillin and three septrin. Amongst the pregnancy and newborn cases five infections resulted in death *in utero*, five early neonatal infection and one maternal infection only. The neonatal cases carried a 20% mortality, all patients being treated with ampicillin plus an aminoglycoside. No maternal deaths were recorded. The local spectrum of disease is similar to that reported in Europe and North America since the late 1960s, however, it is the most complete survey of Listeriosis in one area, recorded in the UK.

### COARCTATION OF THE AORTA IN YOUNG CHILDREN

**G A Russell, P J Berry, K Watterson, J D Wisheart**
Bristol Royal Hospital for Sick Children

Two surgical operations are available for the treatment of coarctation of the aorta: resection with end to end anastomosis or subclavian flap aortoplasty (SFA). A significant number of recurrences have been reported with SFA when performed within the first three months of life. We report the histological findings in 27 coarctation resection specimens from children under three months of age.

A circumferential sling of ductal tissue formed part of the coarctation in 21 cases. In all 21 cases the sling extended distally from the shelf toward the distal resection margin in the form of tongue-like elongations. Excision of these distal extensions of ductal tissue was incomplete in 11 cases.

The refashioned aorta in SFA repairs has a large residual ductal composition. If the subclavian flap does not extend beyond the distal limit of the ductal extension a residual circumferential sling will exist and this may favour restenosis. We have demonstrated that the distal extension may be much longer than is apparent at peroperative inspection. Resection and anastomosis achieves a more complete excision of ductal tissue than the SFA and the circumferential portion is completely removed. If total excision of ductal tissue is central to the prevention of restenosis then an even more radical resection at the distal margin is required.

### A CASE OF MITOCHONDRIAL ENCEPHALOMYOPATHY WITH LACTIC ACIDOSIS AND STROKE-LIKE EPISODES

**J A R Nicoll**
Frenchay Hospital, Bristol

A 14-year old girl presented with an acute episode of left homonymous hemianopia and left hemiparesis with grand mal fits. A CT scan of the brain showed occipital lobe infarction. Two years later she suffered a second similar stroke-like episode. Cerebral angiography was normal. Both episodes were associated with a metabolic acidosis. Approximately a month after the onset of the second episode she passed into a coma and died.

At autopsy there were no significant macroscopic abnormalities outside the brain. In particular the heart and the carotid, vertebral and intracranial arteries were normal. Macroscopic examination of coronal slices of the brain showed thinning of the cortical ribbon in the occipital lobes, and shrinkage and discolouration of the underlying white matter. Diffusely scattered throughout the remainder of the cerebral cortex were multiple focal lesions in which the cortex was granular and discoloured. Histology confirmed the presence of old glial scars in the occipital cortex and showed recent hypoxic/ischaemic lesions elsewhere in the cerebral cortex.

Electron microscopy of skeletal muscle showed that many of the fibres contained abnormally large numbers of mitochondria. The mitochondria showed morphological abnormalities including the presence of filamentous inclusions and large electron dense spherical inclusions. Mitochondria in the cerebral cortex appeared morphologically normal.

We present this as a case of Mitochondrial Encephalomyopathy with Lactic Acidosis and Stroke-like episodes (MELAS). The case is unusual because of the absence of clinically evident muscle weakness. This diagnosis should therefore be thought of in a young person with stroke-like episodes and lactic acidosis even in the absence of apparent muscle weakness. The diagnosis could then be made during life by a muscle biopsy.

### LACTIC DEHYDROGENASE IN THE LOCALIZATION OF URINARY TRACT INFECTION IN CHILDREN: A PRELIMINARY REPORT

**T Humphrey, Hazel Curtis, I Muscat, J D Cruickshank, J Tripp**
Public Health Laboratory, Heavitree, Exeter

A simple test to distinguish those children presenting with UTI who do not require radiology is desirable.

USA studies have shown that a high urinary LDH isoenzyme 5 correctly localized upper UTI in 95% of cases. This appears to be due to a shift in the isoenzymes produced by damaged renal cells. Centrifuging urine within 4 hours of collection and storage at 4° C avoids many technical problems. Freezing at −20° C destroys cathodic LDH isoenzymes, and this property can be used to obviate the need for electrophoresis.

Hospitalized children under seven years were admitted into the study if they had a bacteriologically proven symptomatic UTI, had not had recent antibiotics and were investigated radiologically. Upper UTI (UUTI) was defined by the presence of three or more of: fever 38.5° C; ESR$>$35; CRP$>$20; and decreased renal concentrating ability.

Sixteen children (6 m–7 yrs) were eligible for analysis. In the nine with lower UTI LDH 4+5 levels ranged between 9–100 iu/l (mean 43.8) and in the seven with UUTI between 100–490 iu/l (mean 247). Radiological abnormalities were seen in 4/7 of the latter and 2/9 of the former—a band across the left pelvis (LDH) in one and scars only visible radioisotopically in the other (LDH 64).

In this small series a cut off point of 50 iu/l distinguished those children who had no radiological abnormality. A larger series seems warranted.

### NEUROENDOCRINE CARCINOMA OF THE COLON IN ASSOCIATION WITH VILLOUS ADENOMA

**P Sarsfield, P P Anthony**
Royal Devon and Exeter Hospital, Exeter

Anaplastic tumours of the colon include basaloid or cloacogenic carcinomia of anorectal origin, lymphoma and malignant melanoma. Recently seven cases of ‘neuroendocrine’ carcinoma have been reported in association with villous adenoma which need to be distinguished as their clinical course is different. We wish to describe three further cases that have presented at the Royal Devon and Exeter Hospital.

Two of the patients were male age 88 and 63 years, while the third was female aged 79 years. Two patients presented with a very short history which suggested an appendicular abscess. Laparotomy however in both cases revealed a large tumour in the caecum. The third patient presented with intussusception of the sigmoid colon due to a large tumour. In all three cases the resected bowel specimen showed a large villous adenoma with a separate anaplastic neuroendocrine carcinoma adjacent to but distinct from it. All three patients had both local lymph node and hepatic metastases. Death occurred four, eleven and eighteen weeks after surgery.

Light microscopy of the three tumours showed variable appearance of the solid component. In some areas tumour cells were small, with a high nuclear cytoplasmic ration and resembled ‘oat cell’ carcinoma, whilst elsewhere the nuclei were large and vesicular and the cytoplasm was more abundant. Giant cells were also seen. Immunocytochemistry showed positive staining for CEA, HMFG, CAM 5.2, AE1 and NSE. High molecular weight cytokeratins and AE3 were negative. Electron microscopy was performed on two of the cases and one showed occasional neurosecretory granules. The cytological appearance, neuroendocrine differentiation and positivity for low molecular weight cytokeratins resemble cases published previously in the literature. A highly aggressive course is characteristic of this tumour and all reported patients, including our own, died shortly after presentation with disseminated disease. The association with villous adenoma remains unexplained. Although previously considered rare this cluster of cases may suggest that this highly aggressive neoplasm is more common than thought previously.

